# Supplementary material for: Italian Translation, Cultural Adaptation, and Validation of the Toileting Habit Profile Questionnaire Revised (THPQ-R) in Typically Developing Children: A Cross-Sectional Study
Source: Children (Basel). 2022 Jul 14;9(7):1052. doi: 10.3390/children9071052 (PMC9315688; doi:10.3390/children9071052)
Supplement: Supplementary file 1 [file children-09-01052-s001.zip › children-1722321-supplementary.pdf]

Annex 1

Toileting Habit Profile Questionnaire-Revised<br>Profilo delle abitudini di defecazione<br>Per favore, segnali le risposte che meglio descrivono con che frequenza suo figlio presenta i seguenti comportamenti				
Comportamenti relazionati con il fare la cacca			Frequenza del comportamento<br>1 o 2 punti	
SOR	1	Mio figlio si nasconde per fare cacca.	Frequentemente o sempre	Mai o raramente
SOR	2	Mio figlio mi chiede un pannolino quando ha bisogno di fare la cacca.	Frequentemente o sempre	Mai o raramente
SOR	3	Mio figlio preferisce farsi la cacca addosso nonostante sia vicino al vasino o al water.	Frequentemente o sempre	Mai o raramente
SOR	4	Mio figlio rifiuta di sedersi sul vasino o nel water per fare la cacca peró accetta di sedersi per fare la pipí.	Frequentemente o sempre	Mai o raramente
SOR	5	Mio figlio rifiuta o non si sente a suo agio sedendosi sul water o sul vasino sia per fare pipí che per fare cacca, anche se è a casa sua.	Frequentemente o sempre	Mai o raramente
SOR	6	Mio figlio trattiene la cacca o l'impulso a farla.	Frequentemente o sempre	Mai o raramente
SOR	7	Mio figlio ha delle **abitudini poco comuni** per fare la cacca che implicano **azioni o luoghi** che non sono normalmente associati con il fare la cacca.	Frequentemente o sempre	Mai o raramente

	7 a	Spiegare quali sono le abitudini del suo bambino:		
SOR	8	Mio figlio sembra provare dolore quando fa la cacca, anche se la cacca ha una consistenza morbida.	Frequentemente o sempre	Mai o raramente
SOR	9	Mio figlio si rifiuta di fare cacca in posti che non siano casa sua.	Frequentemente o sempre	Mai o raramente

SOR	10	Mio figlio ha delle reazioni di disgusto per l'odore della sua cacca.	Frequentemente o sempre	Mai o raramente
SOR	11	Mio figlio rifiuta di pulirsi o di essere pulito dopo aver fatto cacca.	Frequentemente o sempre	Mai o raramente
SOR	12	Mio figlio mostra di aver paura o si rifiuta di fare certe cose che normalmente si fanno in bagno come tirare lo sciacquone.	Frequentemente o sempre	Mai o raramente
SOR	13	Mio figlio ha bisogno di distrarsi con qualcosa mentre fa la cacca (con libri, giochi); questo sembra aiutarlo ad accettare di fare la cacca.	Frequentemente o sempre	Mai o raramente
SOR	14	Mio figlio mostra di essere sensibile al gusto o consistenza degli alimenti ricchi in fibre o di farmaci che aiutano a fare la cacca.	Frequentemente o sempre	Mai o raramente
SOR /EP	15	Mio figlio ha iniziato a sentire l'impulso di fare la cacca da molto piccolo (prima dei 12 mesi). Quando mio figlio si lamentava in un certo modo veniva messo sul vasino per fare la cacca.	Frequentemente o sempre	Mai o raramente

SUR /P	16	Mio figlio non sembra sentire lo stimolo di fare la cacca.	Frequentemente o sempre	Mai o raramente
SUR /P	17	Mio figlio non si rende conto che si è sporcato (di cacca) i vestiti o non prova fastidio per essere sporco.	Frequentemente o sempre	Mai o raramente

SOR: iper-reattività sensoriale; SOR/EP: iper-reattività sensoriale/percezione aumentata; SUR/P: ipo-reattività/problematiche di percezione

Calcolare i punti per i primi 15 items. Gli Items 16 e 17 non si includono nel finale

Frequentemente o sempre = 1 punto / Mai o raramente = 2 punti

V7, may 8, 2017

Beaudry-Bellefeuille, Bundy, Lane A, Ramos-Polo & Lane S, 2018
